# Evaluating the Immediate Response of Country-Wide Health Systems to the Covid-19 Pandemic: Applying the Gray Incidence Analysis Model

**DOI:** 10.3389/fpubh.2021.635121

**Published:** 2021-07-15

**Authors:** Tehmina Fiaz Qazi, Muhammad Zeeshan Shaukat, Abdul Aziz Khan Niazi, Abdul Basit

**Affiliations:** ^1^Hailey College of Banking and Finance, University of the Punjab, Lahore, Pakistan; ^2^Faculty of Management Studies, University of Central Punjab, Lahore, Pakistan; ^3^Institute of Business & Management, University of Engineering and Technology, Lahore, Pakistan; ^4^Lahore Institute of Science and Technology, Lahore, Pakistan

**Keywords:** COVID-19 pandemic, deaths, GRA, gray incidence analysis model, health system, tests, Pakistan

## Abstract

The purpose of the study is to evaluate county-wide health systems using the data set of the first wave of the COVID-19 pandemic. The overall design of study comprises a literature review, secondary data, and a mathematical analysis. It is a cross-sectional quantitative study following a deductive approach. It uses the data of the first wave of the COVID-19 pandemic taken from the website of Worldometer as of April 8, 2020. The study uses a gray incidence analysis model (commonly known as Gray Relational Analysis, i.e., GRA) as its research methodology. On the basis of the results of GRA, a classification has been made under a predetermined scheme of ensigns: *much better, better, somewhat better, fair, poor, somewhat worse, and worse* health systems. There are a total 211 countries that have been divided into the seven aforementioned categories. Findings of the study show that Southern Africa Development Community (SADC) countries fall predominantly under the *much better* ensign, whereas Organization for Economic Co-operation and Development (OECD), Schengen Area (SA), and/or European Union (EU) countries fall under the *worse* ensign. Pakistan falls under the ensign of *poor*. It is an original attempt to evaluate the response of health systems based on real data using a scientific methodology. The study provides valuable information about the health systems of the countries for forming an informed opinion about the health systems herein. The study provides useful new information for stakeholders and a new framework for future research.

## Introduction

The COVID-19 pandemic has created serious issues for different countries, particularly those that have weak health systems (1–3). With the outbreak of COVID-19 sustainability, consciousness about healthcare systems has increased, and the need for its performance evaluation has become imperative. The whole world is passing through an abnormal state created by the outbreak of a novel virus COVID-19 from Wuhan, China. Health systems are under extraordinary pressure because of the geometric increase in COVID-19 patients. It is of utmost necessity to evaluate health systems and to revamp them to meet challenges like the current epidemic. The healthcare systems of many countries collapsed during the first wave of COVID-19. It has become obligatory to evaluate the healthcare systems of the world afresh, particularly before embarking on a regime of reforms. The question of measurement of performance and comparison of that performance between healthcare systems of several countries has arisen as an offshoot of the COVID-19 pandemic. Answering this question is not that simple; rather, it is complex and difficult. A plethora of research has already been published on healthcare system in general across the globe, and it is important to document that the efforts have been made by different researchers on many counts, e.g., studies like those on the role of pharmacies in health system of Colombia (4), challenges faced by the national healthcare service in Italy (5), the health system of Mount Sinai, US (6), the proactive role of the public health agency of Canada (1), the strengthening of the Mexican healthcare system by addressing the environmental, social, and healthcare issues (7), the healthcare services of the Hubei province of China (8), the challenges to the Bulgarian healthcare system (9), the resilience of the Taiwanese healthcare system (10), the strained Greek healthcare care system (11), eHealth, remote consultation, and the Australia mental health care setting (12, 13), the resilience of the Spanish healthcare system (14), the strained healthcare system of Latin America (15), a care center in Pakistan (16), the risk to the Brazilian healthcare system (17), the challenges faced by the healthcare system of sub-Saharan Africa (18), and so on. Most of the countries of the world, including Pakistan, are in the process of rethinking their healthcare systems in order to cope with unforeseen epidemics like COVID-19 (19). All countries are introducing rigorous initiatives by way of establishing laboratories, dedicated quarantine facilities, large-scale awareness campaigns, and smart lockdowns to mitigate the proliferation of coronavirus (20). To address the issue of evaluation of health systems affected by the current pandemic, there is a need to develop a methodology to standardize the measurement of health systems of countries concurrently and simultaneously. Warsame et al. (21) asserted that the development of an epidemic response, and an evaluation approach based on a comprehensive evaluation framework needs to be underpinned. To be specific, the following are the research objectives of this study: (i) to evaluate the health systems of the countries using the data set of the first wave of COVID-19 pandemic; (ii) to determine the gray relational grade of countries' health systems; (iii) to group or classify the countries on the bases of the gray relational grade under pre-determined ensigns in order to provide the basis for an informed opinion to discerners; (iv) to discuss the position of selected countries against their regional blocs; (v) to evaluate the position of Pakistan qua rest of the world in general and among Asian countries in particular; and (vi) to discuss the implications for stakeholders. Where does the healthcare system of a certain country rank during the first wave of the COVID-19 pandemic? This is the prime research question this study will address. The authors considered a range of multi-criteria-decision-making techniques: *ANP, FANP, AHP, TOPSIS, DEA, GRA, VIKOR, SWARA, ISM, TISM, MICMAC, SEM*, and *Regression*. Keeping in view the nature of the study, GRA (Gray Incidence Analysis Model) was found to be appropriate since it has the capability to accommodate a large set of cross-sections and a multitude of system variables even with missing, insufficient, and/or incomplete data. Therefore, in this study, the GRA method is used to assess the performance of countries' health systems during the COVID-19 pandemic. It also has the ability to normalize the data having different units of measurement. This study is worthwhile for regulators of health departments, international institutions, frontline soldiers, researchers, political governments, and society at large. The remainder of this paper is arranged as literature review, theoretical framework, methodology, analysis, results and discussion, and concluding remarks.

## Literature Review

There is no dearth of literature on healthcare systems in general, but, in the current panorama of the COVID-19 pandemic, there is a scarcity of peer-reviewed published research on the current situation. However, there is a lot of published/unpublished upcoming literature about the health systems of different countries (22). In this context, the authors have explored the relevant databases like ScienceDirect, Emarald, JStor, Wiley-Blackwell, Taylor & Francis, etc., and have reviewed a significant number of research studies relevant to the phenomenon under study. Highly relevant studies are being reported in order to set the outset of the research: Armocida et al. (5) stated that the National Healthcare Service (responsible for providing health services in regions of Italy) was about to collapse in the Lombardy region of Italy (the most affected region) due to privatization and a €37 billion financial cut over the period of 2010–2019. Chattu et al. (1) revealed that a Canadian public health agency has proved its global health leadership by way of proactive measures taken to address this worldwide COVID-19 outbreak challenge. Chen et al. (8) stressed that pairing assistance (dedicated number of medical personnel to each city depending on the severity of COVID-19) strategy adoption alleviated the pressure on the healthcare system of China, which was a turning point in China's fight against COVID-19. De-Sousa et al. **(author?)** (2) identified 16 physical and mental health challenges being faced by low/middle-income countries and argued that if not addressed, this may get increasingly severe over time. Hsieh (10) argued that Taiwan has taken timely initiatives to mitigate the proliferation of COVID-19, including the activation of the Central Epidemic Command Center (CECC) for communication and coordination, supplying surgical masks, issuing national health insurance cards, and postponing schools' classes. Khan et al. (23) collected data from 302 healthcare workers and proclaimed that the majority of Pakistanis are not well-informed and prepared for the COVID-19 pandemic, and they are also not familiar with the measures to prevent/control contagion. Kim et al. (24) argued that “The University of Washington Medicine's Post-Acute Care Network” established a three-phase approach (initial, delayed, and surge phases) that helped clinics, hospitals, emergency medical services from becoming overwhelmed and to alleviate the spread of COVID-19 cases. Kretchy et al. (25) concluded that retail pharmacies and community pharmacists are easily accessible and are coming forward to share the burden of the healthcare system in low/middle-income countries. Similarly, Amariles et al. (4) revealed an active role of pharmacy staff and community pharmacy to lessen the burden on the healthcare system. Legido-Quigley et al. (26) claimed that Singapore, Hong Kong, and Japan outlined core dimensions for the development of resilience-oriented healthcare systems, including effective intragovernmental coordination, adaptations, allocations of finances, smooth political environment, availability of treatment, supply of medicine, and routine healthcare services. Legido-Quigley et al. (14) revealed that Spanish healthcare systems efficiently managed the first 6 weeks since the first case was identified, but as time passed, pressure built on the six building block of the Spanish healthcare system (i.e., governance, medicine and equipment, financing, healthcare workers, service delivery, and information). Lorenz et al. (27) argued that the outbreak of COVID-19 and dengue fever have caused great damage to the healthcare system in Brazil; alone, COVID-19 has the potential to swamp the Brazilian healthcare system, and a unified partnership between public and private healthcare systems is thus needed to combat this pandemic. Ma et al. (3) identified potential repercussions of the COVID-19 pandemic on health and surgical care in low/middle-income countries and stated that optimizing resources, providing accurate information/knowledge and training to healthcare workers, and protection are the only means to contain the spread of COVID-19. Menon and Padhy (28) revealed that there are some ethical dilemmas faced by healthcare workers even in developed countries and offered some suggestions to trounce them. Mukhtar (29) showed that well-being and mental health care are building blocks of the healthcare system, whereas social distancing/isolation and quarantine are causing potential mental health issues that need to be addressed. Rana et al. (16) explained that, being a lower-middle country, Pakistan has a poor healthcare system wherein the budget allocated to health is only 1% of the GDP. Roder-DeWan (18) argued that low-income countries are hardly able to achieve fewer than half of the elements indispensable for a high-quality healthcare system than that of high-income countries. Telemedicine and telehealth are a fast-emerging concept of health system during the period of COVID-19 to ensure the effectiveness of isolation/social distancing, helping service provision, tracking, tracing, and testing of COVID-19 cases (30–35). After the review of studies like the aforementioned, it has become imperative that we develop a theoretical framework to evaluate healthcare systems at the country level.

## Theoretical Framework

Theories help to explain, predict, understand phenomena, and, sometimes, to challenge or to extend our existing knowledge within the boundaries of given assumptions (36). All that is necessary to use our knowledge and understanding in more informed and effective ways (37). A theoretical framework is used to limit the scope of the relevant data. The selection of a theory depends on its appropriateness, ease of application, and explanatory power. Gray system theory is found to be appropriate in this study keeping in view the objectives of the study and research question under investigation. In order to enhance the clarity and interpretability of results, authors have extended the theoretical framework by way of introducing the system of ensigns. To evaluate the phenomena critically, it is vital to connect to the existing knowledge. The framework also helps to articulate the theoretical assumptions and to identify the limits of results' generalizations. This study uses a theoretical framework to limit the scope of the relevant data by focusing on specific variables and defining them [framework] so that researcher may analyze and interpret the data gathered. The framework also facilitates the understanding of concepts and variables according to given definitions and builds new knowledge by validating or challenging theoretical assumptions (37). The authors have selected the following variables to get on the framework of the study (Table 1).

**Table 1:** Specification of system variables.

**Code**	**Variables**	**Criteria**
1	Total Covid-19 infections	Minimum better
2	New Covid-19 infections	Minimum better
3	Total deaths by Covid-19 infections	Minimum better
4	Total recoveries from Covid-19 infections	Maximum better
5	Active cases of Covid-19	Minimum better
6	Serious/Critical patients of Covid-19	Minimum better
7	Tot cases/1M pop of Covid-19	Minimum better
8	Deaths/1M pop by Covid-19	Minimum better
9	Total tests of Covid-19	Maximum better
10	Tests/1M pop of Covid-19	Maximum better

The variables of social sciences normally have three types of acceptable characteristics. The first type of variable may be *maximum better*, the second type of variable might have characteristics of *minimum better*, and the third type of variable may have characteristics of *target the better*. Close observation of the variables reveals that variables 1,2,3,5,6,7, and 8 possess the characteristic of *minimum better*, whereas variables 4,9, and 10 possess the characteristic of *maximum better*. With this framework, the authors opted to use the Gray Incidence Analysis Model as a solution methodology.

## Methodology

This study follows positivist philosophy and deductive approach. It is a cross-sectional research study that uses data of the first wave of COVID-19 pandemic taken from the website of Worldometer as of April 8, 2020. It uses the Gray Incidence Analysis Model (commonly known as Gray Relational Analysis or simply GRA). It is a unique mathematical approach selected from the array of multi-criteria-decision-making techniques. This technique is frequently employed to use an incomplete and impure set of data for analyzing relations of a multitude of variables. It has prevails on statistical techniques like regression analysis because of their limitations and demand for large amounts of data for generating meager results (38). GRA progresses stepwise (39–43). The first step, in this model, is obtaining data; the second is the creation of a reference series; the third is the generation of a comparable sequence; the fourth is the generation of a reference series; the fifth is the generation of a normalized matrix; the sixth is the calculation of a deviation sequence; the seventh is the creation of absolute values with a difference in the reference sequence and comparable sequence; the eighth is the establishment of a co-efficient matrix of a gray relation system; the ninth is the computation of a gray relational grade; and the tenth step is the arrangement of these in a descending order. The method has been augmented with a classification of the cross-sections using the method of ensigns introduced by the authors. In this method, first, the operational definitions of ensign groups have been generated on the basis of distributing the scale into seven ensigns.

### Applying Gray Incidence Analysis Model

The following steps of GRA were used to access the best performer among different countries of the world.

Step 1: We created a data set (Table 2) and established a decision matrix of data set denoted in the following formula:

(1) $$x_{i}\left(k\right)=\left[\begin{array}{ccc} x_{1}\left(1\right)x_{1}\left(2\right) & \cdots & x_{1}\left(m\right)\\ \vdots & \ddots & \vdots \\ x_{n}\left(1\right)x_{n}\left(2\right) & \cdots & x_{n}\left(m\right) \end{array}\right]$$

**Table 2:** Original country wide data set on corona virus.

**Sr**.	**Country**	**1**	**2**	**3**	**4**	**5**	**6**	**7**	**8**	**9**	**10**
1	Afghanistan	423	0	14	18	391	0	11	0.4	0	0
2	Albania	400	17	22	154	224	7	139	8	2,989	1,039
…	……….	…	…	…	…	…	…	…	…	…	…
…	……….	…	…	…	…	…	…	…	…	…	…
148	Pakistan	4,072	37	58	467	3,547	25	18	0.3	42,159	191
149	Palestine	263	2	1	44	218	0	52	0.2	15,450	3,029
…	……….	…	…	…	…	…	…	…	…	…	…
…	……….	…	…	…	…	…	…	…	…	…	…
210	Zambia	39	0	1	7	31	0	2	0.05	619	34
211	Zimbabwe	11	0	2	0	9	0	0.7	0.1	371	25

*Worldometer (2020)*.

Step 2: We created a reference series and comparison matrix (Table 3) using a classical rule of reference and comparison.

**Table 3:** Reference sequence and comparable sequences.

**Sr**.	**Country**	**Total**	**New**	**Total deaths**	**Total recoveries**	**Active cases**	**Serious/Critical**	**Total Cases/1M pop**	**Deaths/1M pop**	**Total tests**	**Tests/1M pop**
0	Reference sequences	1	0	0	77,279	1	0	0	0	20,82,443	105,458
1	Afghanistan	423	0	14	18	391	0	11	0.4	0	0
2	Albania	400	17	22	154	224	7	139	8	2,989	1,039
…	……….	…	…	…	…	…	…	…	…	…	…
…	……….	…	…	…	…	…	…	…	…	…	…
148	Pakistan	4,072	37	58	467	3,547	25	18	0.3	42,159	191
149	Palestine	263	2	1	44	218	0	52	0.2	15,450	3,029
…	……….	…	…	…	…	…	…	…	…	…	…
…	……….	…	…	…	…	…	…	…	…	…	…
210	Zambia	39	0	1	7	31	0	2	0.05	619	34
211	Zimbabwe	11	0	2	0	9	0	0.7	0.1	371	25

Step 3: We created a normalized matrix (Table 4) using the following formulas for maximum better and minimum better.

**Table 4:** Normalized comparable sequences.

**Sr**.	**Country**	**Total**	**New**	**Total deaths**	**Total recoveries**	**Active cases**	**Serious/Critical**	**Tot Cases/1M pop**	**Deaths/1M pop**	**Total tests**	**Tests/1M pop**
0	Reference sequences	1.00000	1.0000	1.0000	1.00000	1.0000	1.0000	1.0000	1.0000	1.0000	1.0000
1	Afghanistan	0.99895	1.0000	0.9992	0.00023	0.9989	1.0000	0.9987	0.9996	0.0000	0.0000
2	Albania	0.99900	0.9964	0.9987	0.00199	0.9994	0.9992	0.9841	0.9920	0.0014	0.0099
…	……….	…	…	…	…	…	…	…	…	…	…
…	……….	…	…	…	…	…	…	…	…	…	…
148	Pakistan	0.98984	0.9922	0.9966	0.00604	0.9903	0.9973	0.9979	0.9997	0.0202	0.0018
149	Palestine	0.99935	0.9996	0.9999	0.00057	0.9994	1.0000	0.9940	0.9998	0.0074	0.0287
…	……….	…	…	…	…	…	…	…	…	…	…
…	……….	…	…	…	…	…	…	…	…	…	…
210	Zambia	0.99991	1.0000	0.9999	0.00009	0.9999	1.0000	0.9998	1.0000	0.0003	0.0003
211	Zimbabwe	0.99998	1.0000	0.9999	0.00000	1.0000	1.0000	0.9999	0.9999	0.0002	0.0002

For maximum better:

(2) $$x_{i}^{\;*}\left(k\right)=\frac{x_{i}^{\left(0\right)}\left(k\right)-minx_{i}^{\left(0\right)}\left(k\right)}{\max\;x_{i}^{\left(o\right)}\left(k\right)-minx_{i}^{\left(o\right)}\left(k\right)}$$

For minimum better:

(3) $$x_{i}^{\;*}\left(k\right)=\frac{\max\;x_{i}^{\left(o\right)}\left(k\right)-x_{i}^{\left(0\right)}\left(k\right)}{max\;x_{i}^{\left(0\right)}\left(k\right)-minx_{i}^{\left(0\right)}\left(k\right)}$$

For example, for Afghanistan, “*smaller is the better”*

$$\begin{array}{l} x_{1}^{\;*}\left(1\right)=\frac{max\;x_{1}^{0}\left(1\right)-x_{1\;}^{0}\left(1\right)}{max\;x_{1}^{0}\left(1\right)-min\;x_{1}^{o}\left(1\right)}=\;\frac{4005249-423}{4005249-1}\\ \;=0.999895 \end{array}$$

Step 4: We calculated deviation sequences (Table 5) by using the following formula:

(4) $$\Delta_{0\;}\left(\gamma \right)\;=\;\left|x_{0}\left(\gamma \right)-x_{1}\left(\gamma \right)\right|$$

**Table 5:** Deviation sequences.

**Sr**.	**Country**	**Total**	**New**	**Total deaths**	**Total recoveries**	**Active cases**	**Serious/Critical**	**Tot Cases/1M pop**	**Deaths/1M pop**	**Total tests**	**Tests/1M pop**
0	Reference sequences	0.00000	0.00000	0.00000	0.00000	0.00000	0.00000	0.00000	0.00000	0.00000	0.00000
1	Afghanistan	0.00105	0.00000	0.00082	0.99977	0.00107	0.00000	0.00126	0.00040	1.00000	1.00000
2	Albania	0.00100	0.00358	0.00128	0.99801	0.00061	0.00076	0.01591	0.00798	0.99856	0.99015
…	……….	…	…	…	…	…	…	…	…	…	…
…	……….	…	…	…	…	…	…	…	…	…	…
148	Pakistan	0.01016	0.00779	0.00339	0.99396	0.00969	0.00273	0.00206	0.00030	0.97976	0.99819
149	Palestine	0.00065	0.00042	0.00006	0.99943	0.00059	0.00000	0.00595	0.00020	0.99258	0.97128
…	……….	…	…	…	…	…	…	…	…	…	…
…	……….	…	…	…	…	…	…	…	…	…	…
210	Zambia	0.00009	0.00000	0.00006	0.99991	0.00008	0.00000	0.00023	0.00005	0.99970	0.99968
211	Zimbabwe	0.00002	0.00000	0.00012	1.00000	0.00002	0.00000	0.00008	0.00010	0.99982	0.99976

For example, for Albania

$$\Delta_{02}\;\left(2\right)\;=\;\left|x_{0}^{*}\left(2\right)-x_{2}^{*}\left(2\right)\right|=\;\left|1-0.9964\right|=0.0036$$

Step 5: The Gray relational co-efficient is calculated (Table 6) by using the following formula based on values of normalized sequences. Term ξis the distinguishing co-efficient between 0 and 1, the usual value of which is 0.5 in literature.

(5) $$\begin{array}{l} \gamma \left[x_{0}^{\;*}\left(k\right),x_{i}^{*}\left(k\right)\right]=\frac{\Delta_{min\;}+\xi \;\Delta_{\max}}{x_{0i}\left(k\right)+\xi \;\Delta_{\max}}\;,\;0<\gamma \left[x_{o}^{*}\left(k\right),x_{i}^{*}\left(k\right)\right]\\ \;\leq 1 \end{array}$$

**Table 6:** Gray relational co-efficient.

**Sr**.	**Country**	**Total**	**New**	**Total deaths**	**Total recoveries**	**Active cases**	**Serious/Critical**	**Tot Cases/1M pop**	**Deaths/1M pop**	**Total tests**	**Tests/1M pop**
0	Reference Sequences	1.00000	1.00000	1.00000	1.00000	1.00000	1.00000	1.00000	1.00000	1.00000	1.00000
1	Afghanistan	0.99790	1.00000	0.99837	0.33339	0.99787	1.00000	0.99749	0.99920	0.33333	0.33333
2	Albania	0.99801	0.99289	0.99744	0.33378	0.99878	0.99848	0.96917	0.98428	0.33365	0.33554
…	……….	…	…	…	…	…	…	…	…	…	…
…	……….	…	…	…	…	…	…	…	…	…	…
148	Pakistan	0.98008	0.98465	0.99327	0.33468	0.98099	0.99458	0.99590	0.99940	0.33789	0.33374
149	Palestine	0.99869	0.99916	0.99988	0.33346	0.99882	1.00000	0.98824	0.99960	0.33499	0.33984
…	……….	…	…	…	…	…	…	…	…	…	…
…	……….	…	…	…	…	…	…	…	…	…	…
210	Zambia	0.99981	1.00000	0.99988	0.33335	0.99984	1.00000	0.99954	0.99990	0.33340	0.33340
211	Zimbabwe	0.99995	1.00000	0.99977	0.33333	0.99996	1.00000	0.99984	0.99980	0.33337	0.33339

For example, for Albania,

$$\begin{array}{l} \gamma \left[x_{0}^{*}\left(2\right),\;x_{2}^{*}\left(2\right)\right]=\;\frac{\Delta_{min\;+\xi }\Delta_{\max}}{\Delta_{2}\left(2\right)+\xi_{\max}}=\frac{0+\left(0.5\right)\times 1}{0.0036+\left(0.5\right)\times 1}\\ \;=0.9928 \end{array}$$

Step 6: The weighted sum of gray relational co-efficient (Gray Relational Grade) is calculated (Table 7) by using the following formula:

(6) $$\gamma \left(x_{0}^{\;*},x_{i}^{*}\right)=\;\sum_{k=1}^{n}\beta_{k}\text{γ }\left[x_{0}^{\;*}\left(k\right),x_{i}^{\;*}\left(k\right)\right]$$

(7) $$\sum_{k=1}^{n}\beta_{k}=1$$

**Table 7:** Gray relational grades.

**Sr**.	**Country**	**Gray relational grade**
0	Reference sequences	1.0000
1	Afghanistan	0.7991
2	Albania	0.7942
…	……….	…
…	……….	…
148	Pakistan	0.7935
149	Palestine	0.7993
…	……….	…
…	……….	…
210	Zambia	0.7999
211	Zimbabwe	0.7999

For example, for Albania,

$$\begin{array}{l} \text{γ}\left(x_{0}^{\;*},x_{2}^{*}\right)=\sum_{k=1}^{n}\beta_{k}\;\gamma \left[x_{0}^{\;*}\left(2\right),x_{2}^{\;*}\left(k\right)\right]\\ =0.10\times \;\left(\begin{array}{c} 0.9980+0.9929+0.9974+0.3338+0.9988\\ +0.9985+0.9692+0.9843+0.3337+0.3355 \end{array}\right)\\ =0.7942 \end{array}$$

The authors have introduced the method of ensigns to represent the gray relational ranks of the countries. The ensigns were taken on the basis of the pattern of the ordinal scale, including *much better, better, somewhat better, fair, poor, somewhat worse*, and *worse*. The operational definitions of these ensigns are given in Table 8. This method has been introduced to logically represent and interpret the results of gray relational analysis particularly that of the ranks of the countries qua other counterparts. This also facilitates the provision of insight into the different blocs of countries currently existing in the world. In fact, there are 211 total countries under investigation and the scale of ensigns consists of seven items, therefore, ~30 countries are categorized in each bracket of an ensign. The bracket of gray relational grade has also been mentioned against each scale item to make the information more objective and meaningful.

**Table 8:** Scheme of grouping the countries under different ensigns on the basis of gray relational grades of health systems.

**Sr**.	**Ensign**	**Description**
1	Much better	Countries having a gray relational grade ranging from 0.8203 to 0.7999 are considered as having an excellent health system (top thirty countries).
2	Better	Countries having a gray relational grade ranging from 0.7999 to 0.7994 are considered as having a very good health system.
3	Somewhat better	Countries having a gray relational grade ranging from 0.7994 to 0.7980 are considered as having a good health system.
4	Fair	Countries having a gray relational grade ranging from 0.7978 to 0.7947 are considered as having a satisfactory health system.
5	Poor	Countries having a gray relational grade ranging from 0.7945 to 0.7890 are considered as having a weak health system.
6	Somewhat worse	Countries having a gray relational grade ranging from 0.7889 to 0.7724 are considered as having a very weak health system.
7	Worse	Countries having a gray relational grade ranging from 0.7723 to 0.4854 are considered as having the worst health system.

Readers will find ensigns information significantly helpful in making an informed opinion about a countries' and/or blocs' health systems.

## Results and Discussion

### Results

We measured the performance of healthcare systems in countries and compared those performances with others as an offshoot of the COVID-19 pandemic. This is important because the countries are planning to revisit the architecture of their healthcare systems, and the answer is not that simple. The healthcare systems of many countries collapsed as a result of the first wave of COVID-19, and, therefore, it is vital to evaluate health systems before any revamping. Hence the aim of this study is to evaluate healthcare systems in different countries, including Pakistan, and compare them against each other. The study uses Gray Relational Analysis (GRA) as its methodology to evaluate the system and it uses secondary data from the website of Worldometer (44). The study thus provides understanding to readers in terms of the capability of healthcare systems in different countries in responding to pandemics like COVID-19. The authors gathered a significant number of articles, reports, statistical bulletins, and official documents from authoritative websites and examined the findings to set the context of the study. Results of the analysis are given in Table 9.

**Table 9:** Results of gray relational analysis.

**Country**	**Gray relational grades**	**Rank**	**Country**	**Gray relational grades**	**Rank**	**Country**	**Gray relational grades**	**Rank**
Reference sequences	1.0000	0	Maldives	0.7992	70	Greece	0.7910	141
**Much better**			Suriname	0.7992	71	North Macedonia	0.7909	142
Faeroe Islands	0.8203	1	Jordan	0.7992	72	Turks and Caicos	0.7909	143
Vietnam	0.8010	2	Belize	0.7991	73	Bosnia and Herzegovina	0.7909	144
China	0.8008	3	Afghanistan	0.7991	74	Armenia	0.7908	145
New Caledonia	0.8004	4	Hong Kong	0.7989	75	Moldova	0.7904	146
Bhutan	0.8002	5	Burkina Faso	0.7989	76	Kuwait	0.7898	147
UAE	0.8002	6	Greenland	0.7988	77	Singapore	0.7894	148
Nepal	0.8000	7	El Salvador	0.7987	78	India	0.7893	149
Papua New Guinea	0.8000	8	Azerbaijan	0.7987	79	Belarus	0.7890	150
South Sudan	0.8000	9	Kazakhstan	0.7986	80	**Somewhat worse**		
Mozambique	0.8000	10	Cameroon	0.7986	81	Philippines	0.7889	151
Burundi	0.8000	11	St. Vincent Grenadines	0.7985	82	Guadeloupe	0.7889	152
Somalia	0.8000	12	Macao	0.7984	83	Martinique	0.7888	153
Timor-Leste	0.8000	13	Cuba	0.7984	84	Saudi Arabia	0.7886	154
Chad	0.8000	14	Caribbean Netherlands	0.7984	85	Falkland Islands	0.7884	155
Uganda	0.8000	15	Uzbekistan	0.7983	86	Aruba	0.7883	156
MS Zaandam	0.8000	16	Bolivia	0.7983	87	Dominican Republic	0.7882	157
Tanzania	0.8000	17	Saint Lucia	0.7983	88	Croatia	0.7881	158
Botswana	0.8000	18	South Africa	0.7981	89	Ukraine	0.7881	159
Sudan	0.7999	19	Georgia	0.7980	90	St. Barth	0.7878	160
CAR	0.7999	20	**Fair**			Serbia	0.7875	161
Myanmar	0.7999	21	Brunei	0.7978	91	Mayotte	0.7867	162
Malawi	0.7999	22	Iraq	0.7978	92	Malaysia	0.7863	163
Zimbabwe	0.7999	23	Honduras	0.7978	93	Indonesia	0.7859	164
Angola	0.7999	24	British Virgin Islands	0.7978	94	Slovenia	0.7858	165
Sierra Leone	0.7999	25	Slovakia	0.7978	95	Cayman Islands	0.7851	166
Laos	0.7999	26	Guyana	0.7977	96	Ecuador	0.7834	167
Mauritania	0.7999	27	Grenada	0.7976	97	Chile	0.7833	168
Nicaragua	0.7999	28	Egypt	0.7975	98	Czechia	0.7830	169
Syria	0.7999	29	Seychelles	0.7975	99	Bermuda	0.7825	170
Zambia	0.7999	30	Bangladesh	0.7973	100	Iceland	0.7825	171
**Better**			Costa Rica	0.7973	101	Poland	0.7821	172
Haiti	0.7999	31	Kyrgyzstan	0.7972	102	Estonia	0.7811	173
Benin	0.7999	32	Bahrain	0.7971	103	Mexico	0.7811	174
Namibia	0.7999	33	Trinidad and Tobago	0.7971	104	Finland	0.7796	175
Taiwan	0.7999	34	Curaçao	0.7970	105	Qatar	0.7794	176
Equatorial Guinea	0.7999	35	French Polynesia	0.7968	106	Panama	0.7764	177
Gambia	0.7999	36	Bulgaria	0.7967	107	Saint Martin	0.7745	178
Libya	0.7999	37	Uruguay	0.7966	108	Norway	0.7738	179
Western Sahara	0.7998	38	Dominica	0.7963	109	Montserrat	0.7724	180
Mongolia	0.7998	39	Tunisia	0.7963	110	**Worse**		
Cambodia	0.7998	40	Saint Kitts and Nevis	0.7962	111	Isle of Man	0.7723	181
Ethiopia	0.7998	41	Saint Pierre Miquelon	0.7962	112	Russia	0.7715	182
Eswatini	0.7998	42	Djibouti	0.7957	113	Romania	0.7708	183
Mali	0.7998	43	Oman	0.7956	114	Brazil	0.7702	184
Liberia	0.7998	44	Anguilla	0.7956	115	Liechtenstein	0.7690	185
Eritrea	0.7998	45	Colombia	0.7955	116	Gibraltar	0.7689	186
Rwanda	0.7997	46	Lebanon	0.7955	117	Canada	0.7679	187
Togo	0.7997	47	Argentina	0.7949	118	Israel	0.7641	188
Nigeria	0.7997	48	Bahamas	0.7948	119	Monaco	0.7635	189
Madagascar	0.7996	49	Mauritius	0.7947	120	Channel Islands	0.7631	190
Sao Tome and Principe	0.7996	50	**Poor**			Ireland	0.7620	191
Guinea	0.7996	51	Latvia	0.7945	121	Sint Maarten	0.7610	192
Guatemala	0.7996	52	French Guiana	0.7944	122	Denmark	0.7574	193
Fiji	0.7996	53	Morocco	0.7943	123	Austria	0.7495	194
Gabon	0.7996	54	Albania	0.7942	124	Luxembourg	0.7437	195
Guinea-Bissau	0.7996	55	New Zealand	0.7940	125	Vatican City	0.7333	196
Congo	0.7995	56	Algeria	0.7940	126	Turkey	0.7319	197
DRC	0.7995	57	Australia	0.7939	127	Portugal	0.7301	198
Venezuela	0.7995	58	Pakistan	0.7935	128	Sweden	0.7221	199
Senegal	0.7995	59	Barbados	0.7935	129	Andorra	0.7061	200
Diamond Princess	0.7994	60	Japan	0.7932	130	Switzerland	0.7030	201
**Somewhat better**			Hungary	0.7925	131	San Marino	0.6712	202
Kenya	0.7994	61	S. Korea	0.7925	132	Germany	0.6709	203
Ghana	0.7994	62	Thailand	0.7923	133	Netherlands	0.6681	204
Niger	0.7993	63	Peru	0.7923	134	UK	0.6630	205
Sri Lanka	0.7993	64	Malta	0.7922	135	Belgium	0.6494	206
Ivory Coast	0.7993	65	Antigua and Barbuda	0.7919	136	Iran	0.6255	207
Cabo Verde	0.7993	66	Cyprus	0.7918	137	USA	0.5785	208
Jamaica	0.7993	67	Lithuania	0.7916	138	France	0.5773	209
Palestine	0.7993	68	Réunion	0.7912	139	Italy	0.5661	210
Paraguay	0.7992	69	Montenegro	0.7911	140	Spain	0.4854	211

Using the gray relational analysis (i.e., mathematical technique of data analysis with the capability of handling a multitude of variables, cases, and time periods), the study has characterized 211 countries of the world into seven different categories (Table 8). From the result of GRA, it can be learned that there are a total of 30 countries categorized as countries having a *much better* healthcare system, most of which are member countries of the Southern Africa Development Community (SADC); 30 countries are under the *better* ensign, most of which are member countries of the West African Economic and Monetary Union (WAEMU); 30 are under the ensign of *somewhat better*, most of which are member countries of Caribbean Community and Common Market (CARICOM); 30 are under the ensign of *fair*, most of which are member countries of Arabian Countries (AC); 30 are under the ensign of *poor*, most of which are member countries of Organization for Economic Co-operation and Development (OECD); 30 are under the ensign of *somewhat worse*, most of which are member countries of the Organization for Economic Co-operation and Development (OECD); and 30 are under the ensign of *worse*, most of which are member countries of the Organization for Economic Co-operation and Development (OECD), Schengen Area (SA), and/or European Union (EU). Pakistan fall under the ensign of *poor*, therefore have a weak health system.

### Discussion

The purpose of the study is to evaluate the health systems at the country level using GRA. The results are classified under a predetermined scheme of ensigns. It is different on many counts from what contemporary literature says in terms of the composite measurement matrix, number of countries, methodology, data set, context, and classification. Traditional studies usually provide statistical analysis with very limited insights. This finding is consistent with on-ground realities. From the result of the study, it can be learned that the healthcare system of advanced countries, i.e., UK, USA, France, Denmark, etc. (almost whole western Europe/Schengen area/OECD), has a very poor response to the shock of COVID-19 pandemic, which is in contrast to the myth that these countries have the best healthcare systems in the world. In this way, the result of the study provides some evidence that it is the other way around. Pakistan's healthcare system, though poor, still ranks above most of the advanced countries as far as the response to the first shock of the COVID-19 pandemic is concerned (Table 9).

## Concluding Remarks

With the outbreak of COVID-19, consciousness about the sustainability of healthcare systems has increased, and there has been a marked call for the need to evaluate its performance. The whole world is passing through an abnormal condition created with the outbreak of the novel coronavirus. Healthcare systems are under extraordinary pressure. It is of utmost necessity to evaluate healthcare systems and to revamp them to meet challenges like the current epidemic. The healthcare systems of many countries collapsed during the first wave of COVID-19. It has become imperative to evaluate the healthcare systems of the world afresh, particularly before embarking on the regime of any reforms. The purpose of the study was to evaluate the health systems of all countries. The study also aimed to evaluate Pakistan's healthcare system against that of the rest of the world. The overall design of the study comprises literature reviews, secondary data, and mathematical analysis. It is a cross-sectional quantitative study following a deductive approach. The study uses Gray Relational Analysis (GRA) as its research methodology. The findings of the study show that there are 30 countries categorized as countries having *much better* health systems, most of which are member countries of the Southern Africa Development Community (SADC); 30 under the *better* ensign, most of which are member countries of West African Economic and Monetary Union (WAEMU); 30 are under the ensign of *somewhat better*, most of which are member countries of the Caribbean Community and Common Market (CARICOM); 30 are under the ensign of *fair*, most of which are member countries of Arabian Countries (AC); 30 are under the ensign of *poor*, most of which are member countries of the Organization for Economic Co-operation and Development (OECD); 30 are under the ensign of *somewhat worse*, most of which are member countries of Organization for Economic Co-operation and Development (OECD), and 30 are under the ensign of *worse*, most of which are member countries of the Organization for Economic Co-operation and Development (OECD), Schengen Area (SA), and/or European Union (EU). Pakistan falls under the ensign of *poor* and therefore has a weak healthcare system. The study revealed several practical and theoretical implications. The study has made several contributions to existing literature. It contributes firsthand information about healthcare systems, such as where a country stands as against reference values. It contributed gray relational grades and ranks assigned to every country using a multitude of variables. It also contributed by way of classification of healthcare systems into groups under different ensigns to making the results more simple. It provides a potential framework to guide academics and practitioners for future research. The study improves the understanding of concerned people about healthcare systems. Regulators and management can gain understanding from this study for policy decisions. The study builds awareness on systemic issues. The study also has some limitations, and it is worthwhile to mention these limitations in order to achieve clarity. Firstly, it is a cross-sectional study, and future studies may be longitudinal, using time series/panel data. Secondly, the study used a data set from the Worldometer website as of April 8, 2020; therefore, the generalizability of results is limited accordingly. Future studies may use different data sets (e.g., data of the WHO, WDI, etc.) in the same theoretical scheme to confirm/validate/substantiate the results. Thirdly, this study uses GRA the hierarchicalization technique, and there are other techniques for this purpose as well, e.g., RIDIT, AHP, TOPSIS, SWARA, VIKOR, and ISM, and future studies may thus use these methodologies. Finally, we have given equal weight to all variables; this may be changed, and future researchers may use AHP, expert opinions, or the entropy method.

## Data Availability Statement

The raw data supporting the conclusions of this article will be made available by the authors, without undue reservation.

## Author Contributions

MS initiated the idea and worked on gray analysis. TQ worked on the relevant literature of the topic. AK collected the data and performed the analyses. AB worked on the write up. All authors contributed to the article and approved the submitted version.

## Conflict of Interest

The authors declare that the research was conducted in the absence of any commercial or financial relationships that could be construed as a potential conflict of interest.
